# Use of quantitative T2 mapping for the assessment of renal cell carcinomas: first results

**DOI:** 10.1186/s40644-019-0222-8

**Published:** 2019-06-07

**Authors:** Lisa C. Adams, Keno K. Bressem, Phillipp Jurmeister, Ute L. Fahlenkamp, Bernhard Ralla, Guenther Engel, Bernd Hamm, Jonas Busch, Marcus R. Makowski

**Affiliations:** 1 Department of Radiology, Charité, Charitéplatz 1, 10117 Berlin, Germany; 2 Department of Radiology, Charité, Hindenburgdamm 30, 12203 Berlin, Germany; 3 Department of Pathology, Charité, Charitéplatz 1, 10117 Berlin, Germany; 4 Department of Urology, Charité, Charitéplatz 1, 10117 Berlin, Germany

**Keywords:** MRI mapping techniques, Quantitative MRI, T2 mapping, Clear cell renal cell carcinoma, Tumor grading

## Abstract

**Background:**

Correct staging and grading of patients with clear cell renal cell carcinoma (cRCC) is of clinical relevance for the prediction of operability and for individualized patient management. As partial or radial resection with postoperative tumor grading currently remain the methods of choice for the classification of cRCC, non-invasive preoperative alternatives to differentiate lower grade from higher grade cRCC would be beneficial.

**Methods:**

This institutional-review-board approved cross-sectional study included twenty-seven patients (8 women, mean age ± SD, 61.3 ± 14.2) with histopathologically confirmed cRCC, graded according to the International Society of Urological Pathology (ISUP). A native, balanced steady-state free precession T2 mapping sequence (TrueFISP) was performed at 1.5 T. Quantitative T2 values were measured with circular 2D ROIs in the solid tumor portion and also in the normal renal parenchyma (cortex and medulla). To estimate the optimal cut-off T2 value for identifying lower grade cRCC, a Receiver Operating Characteristic Curve (ROC) analysis was performed and sensitivity and specificity were calculated. Students’ t-tests were used to evaluate the differences in mean values for continuous variables, while intergroup differences were tested for significance with two-tailed Mann-Whitney-U tests.

**Results:**

There were significant differences between the T2 values for lower grade (ISUP 1–2) and higher grade (ISUP 3–4) cRCC (*p* < 0.001), with higher T2 values for lower grade cRCC compared to higher grade cRCC. The sensitivity and specificity for the differentiation of lower grade from higher grade tumors were 83.3% (95% CI: 0.59–0.96) and 88.9% (95% CI: 0.52–1.00), respectively, using a threshold value of ≥110 ms. Intraobserver/interobserver agreement for T2 measurements was excellent/substantial.

**Conclusions:**

Native T2 mapping based on a balanced steady-state free precession MR sequence might support an image-based distinction between lower and higher grade cRCC in a two-tier-system and could be a helpful addition to multiparametric imaging.

**Electronic supplementary material:**

The online version of this article (10.1186/s40644-019-0222-8) contains supplementary material, which is available to authorized users.

## Background

Globally, renal cell carcinomas (RCC) have significant impact with approximately 65,000 new cases in the United States and 84,000 cases in the European Union each year [1, 2], with clear cell renal cell carcinomas (cRCC) being the most common subtype [3, 4]. Given that lower grade cRCC carry a significantly better prognosis than higher grade cRCC, it would be desirable to individualize treatment options, which could entail a radical approach for higher grade cRCC and a more conservative management such as active surveillance for lower grade cRCC [5]. Currently, partial or radial resection with postoperative tumor grading remains the method of choice for the classification of cRCC, because image-based biopsies or fine needle aspiration remain controversial due to their invasiveness, sampling errors, and risk of needle tract seeding [6]. Therefore, non-invasive preoperative alternatives to differentiate lower grade from higher grade cRCC would be beneficial [6].

Magnetic resonance imaging (MRI) is a non-invasive imaging modality, not relying on ionizing radiation. T2 weighted MRI sequences allow for visualization of inflammation and edema [7]. While conventional MRI only enables a qualitative image interpretation based on signal intensity analysis with arbitrary units, T2 mapping with voxel-wise evaluation of proton spin-spin relaxation times allows for a non-invasive visualization and quantification of tissue composition [8]. As quantitative T2 values reflect tissue composition and, in particular, free water content, T2 mapping is sensitive to tissue hydration or edema without the need for contrast agents and thus shows the potential to become a ‘non-invasive biopsy’ [9, 10].

While initially developed in the context of cardiac imaging, e.g. for quantification of myocardial edema as an early predictor of myocardial injury [7], T2-weighted parametric mapping techniques are increasingly applied in other organs, such as the liver or kidney, providing a pixel-by-pixel map of various T2 relaxation times [11–13].

In the context of brain tumors, it was previously suggested that tumors with higher cellularity showed a corresponding reduction in the extracellular fluid space [14]. While lower grade cRCC are associated with small nucleoli and low nuclear-to-cytoplasmic ratios, higher grade cRCC are characterized by nuclear polymorphism, higher cellularity and nuclear-to-cytoplasmic ratios [15], whereby it can be assumed that the extracellular fluid is subsequently decreased.

We hypothesized, that quantitative T2 mapping could be used to distinguish lower from higher grade cRCC by visualization of differences in tissue composition, e.g. extracellular liquid. This proof-of-concept study therefore investigates the feasibility of a T2 mapping approach to grade cRCC, correlating imaging findings with the corresponding histology.

## Methods

### Study population

This cross-sectional study was approved by the local Institutional Review board with written consent obtained from all participants prior to examinations. Between January 2017 and October 2018, 42 consecutive patients with suspected RCC, who agreed to participate, had no previous ablations or contraindications to MRI, were referred to our department for abdominal MRI. Out of these, 27 patients with histologically validated cRCC were included in the final analysis. The exclusions were as follows: 3 patients with urothelial carcinomas, 2 patients with oncocytomas, 2 patients with atypical angiomyolipomas, 2 patients without histologic examination (missing data on the reference standard), 4 patients with incorrect acquisition protocols and 2 patients with insufficient image quality due to motion artifacts.

### Imaging protocol

Image acquisitions were performed using a 1.5 T clinical MRI scanner (Avanto; Siemens Medical Solutions, Erlangen, Germany) with dedicated 18-channel body and spine matrix coils. Apart from a clinical routine image protocol of the kidneys, the patients received a native, balanced steady-state free precession T2 mapping sequence (T2-prepared single-shot TrueFISP) in coronal plane, which was adjusted to the long axis of the kidney. The routine imaging protocol of the kidney included a coronal/sagittal T2 half Fourier single-shot turbo spin echo sequence (HASTE), a native coronal 3D gradient echo pulse T1-weighted (FLASH) sequence, a T1-FLASH angiography (contrast agent: gadoterate meglumine (Dotarem®, Guerbet, France), and a delayed fat saturated 3D T1 VIBE (volumetric interpolated breath-hold examination) sequence. Bolus tracking was used to determine the first pass and 3D FLASH images were obtained in the corticomedullary and nephrogenic phase. The T2-preparation was an iterative Carr-Purcell Malcom-Levitt (MLEV) sequence [16, 17]. T2 prepared-TrueFISP images were acquired at intervals of 3 interbeat/RR intervals with simulation of the electrocardiogram with 1 s per beat to allow for sufficient magnetization recovery in between acquisitions. For each image, the acquisition window was set in the same diastolic phase. The time of acquisition for the TrueFISP sequence was 14 s per slice acquisition, whereby T2 maps were acquired in three slices. All T2 maps were acquired prior to application of contrast agents. Please refer to Table 1 for detailed imaging parameters.

**Table 1:** Tabulated overview of MR imaging parameters

Sequence	T2-HASTE	True FISP*	T1 3D-FLASH*
Scan plane	Coronal	Coronal	Coronal
Voxel size (mm)	1.7 × 1.3 × 5.0	1.6 × 1.6 × 4.0	1.6 × 1.0 × 1.4
Number of slices	25	1	1
Slice thickness (mm)	5	4	1.40
TR/ TE (ms)	800/ 89	634.3/ 1.18	2.88/0.98
Averages	1	4	1
FoV (mm)	400	400	500
Flip angle (°)	170	70	25
Matrix	320	256	512
Bandwidth (Hz/Px)	422	930	440
Fat saturation	None	None	Yes
Parameter map type	–	T2 map (2D)	–
Number of T2 preps (lengths in ms)	–	3 (0; 24; 55)	–
Echo spacing (ms)	–	2.8	–
Phase encoding direction	R> > L	R> > L	R> > L

Descriptions: *FISP: Fast imaging with steady-state free frecession. **FLASH: Fast low-angle shot

T2 maps were automatically calculated on a pixel-by-pixel basis, based on the assumption of mono-exponential signal decay. They were displayed by a customized 12-bit lookup table with a visible colour map, whereby the signal intensity of each pixel reflected its absolute T2 value.

### Imaging evaluation

PACS workstations (centricity radiology; GE Healthcare) were used for image evaluation/analysis. An evaluation of T2 mapping images was performed independently by two radiologists, who were blinded to the histopathological findings, enabling the assessment of interobserver agreement. One radiologist repeated the measurements after 2 weeks and two further ROI measurements were conducted after 3 months to enable a comprehensive intraobserver assessment. In large tumors with necrosis zones, circular 2D ROIs were placed within the most homogeneous and bright appearing portion of solid tumor area on the basis of visual assessment (in the postcontrast sequences), also in conjunction with T2-weighted images and were set in as large an area as possible. In small tumors without apparent necrosis zones, a circular 2D ROI was drawn around the entire tumor to avoid measurement inaccuracies (for illustration of exemplary measurements refer to Additional file 1: Figure S1). The respective ROIs were then copied to the TrueFISP (T2 mapping) sequence, using a semi-automatic co-registration tool. In case of breathing or motion artefacts, an additional visual correction was applied. As T2 maps were acquired within a single breathhold, there was not motion between the different T2¬ weighted images. In all cases, it was taken care not to include the normal renal cortex, perinephric or sinus fat within the measured ROIs. Regions of necrosis and cystic degeneration were avoided and identified by lack of enhancement on postcontrast images [18, 19]. In one patient, who did not receive postcontrast imaging due to severe renal insufficiency, the initial 2D ROI was drawn in the T2 HASTE image in the most solid and homogeneous appearing tumor portion and then copied to the TrueFISP sequence. To measure the T2 values of renal cortex and medulla, 2D ROIs were placed in a healthy portion of the renal cortex and medulla, avoiding positioning on the boundary between cancerous and normal parenchyma. In case of extensive tumor infiltration of the kidney (which was the case in two patients), ROI measurements of the renal cortex and medulla were performed in the contralateral heathy kidney

### ISUP grading

Resected cRCC specimens were examined by a pathologist. They were then classified into four levels by the International Society of Urological Pathology (ISUP)/ World Health Organization (WHO) [12]. Based on the assessment of nuclear prominence, Grade 1 is defined by inconspicuous/missing nuclei at × 400 magnification. For grade 2, the nuclei are clearly visible at a magnification of × 400 and for grade 3, the nuclei are visible at a magnification of × 100. Finally, grade 4 tumors are highly polymorphic, with rhomboid and/or sarcomatoid differentiation [12].

### Statistical analysis

All statistical analysis was performed with the statistical software “R” (Version 3.2.2, R Development Core Team, 2015). Variables were averaged across measurements and expressed as mean ± standard deviations in case of normal distribution and with median and interquartile range in absence of normal distribution. Students’ t-tests were used to evaluate the differences in mean values for continuous variables, while intergroup differences were tested for significance using the two-tailed Mann-Whitney-U test. Boxplots were created to show the distribution of the averaged quantitative T2 values among the different grades. To estimate the optimal cut-off T2 value for identifying a lower grade cRCC, a Receiver Operating Characteristic Curve (ROC) analysis was performed with exploratory selection of the optimal cut-off value. Sensitivity and specificity were calculated and based on values averaged over the two observers. Intraobserver and interobserver agreement were calculated using Bland Altman plots with limits of agreement and corresponding confidence intervals, whereby for the intraobserver agreement a graphical method for assessment of more than two readings was used [20]. In addition, the Coefficient of Variation (CoV) and the intraclass coefficient (ICC) were calculated. For the ICC, reliability was defined as excellent for values above 0.9, as good for values between 0.75 and 0.90, as moderate for 0.5–0.75 and as poor for values below 0.5 [21]. A *p*-value < 0.05 was considered to indicate a significant difference.

## Results

**Fig. 1:**
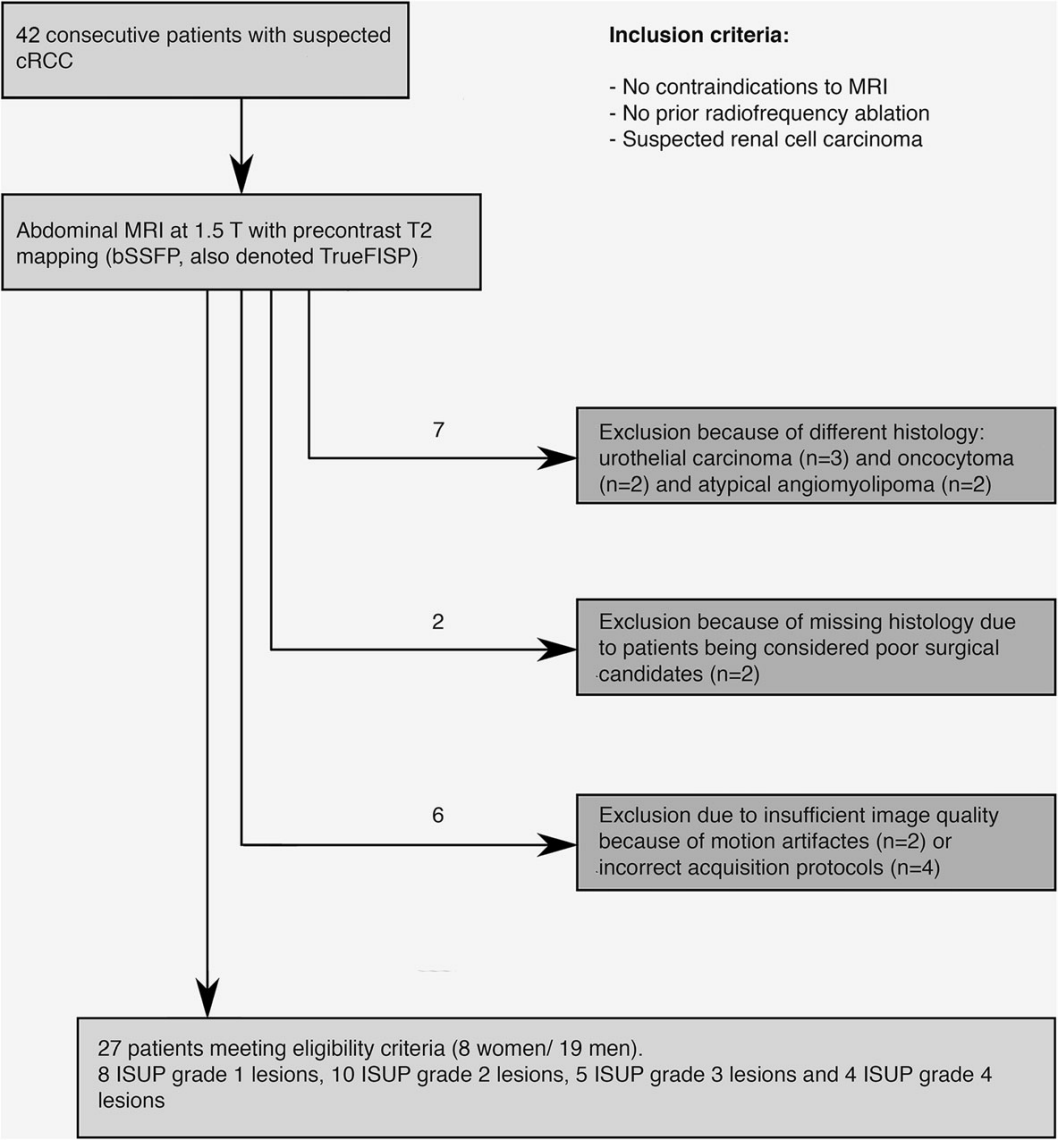
Study workflow. Diagram illustrating the workflow of study participants and showing the reasons for exclusion as well as the final study population, meeting the eligibility criteria

The final study population consisted of 27 patients (19 men, 8 women, mean age, 61.3 ± 14.2; age range, 34–87 years) with histologically diagnosed cRCC. Figure 1 provides a study workflow and Table 2 gives an overview of the study characteristics. 14 lesions were in the left kidney and 13 lesions were located in the right kidney.

**Fig. 2:**
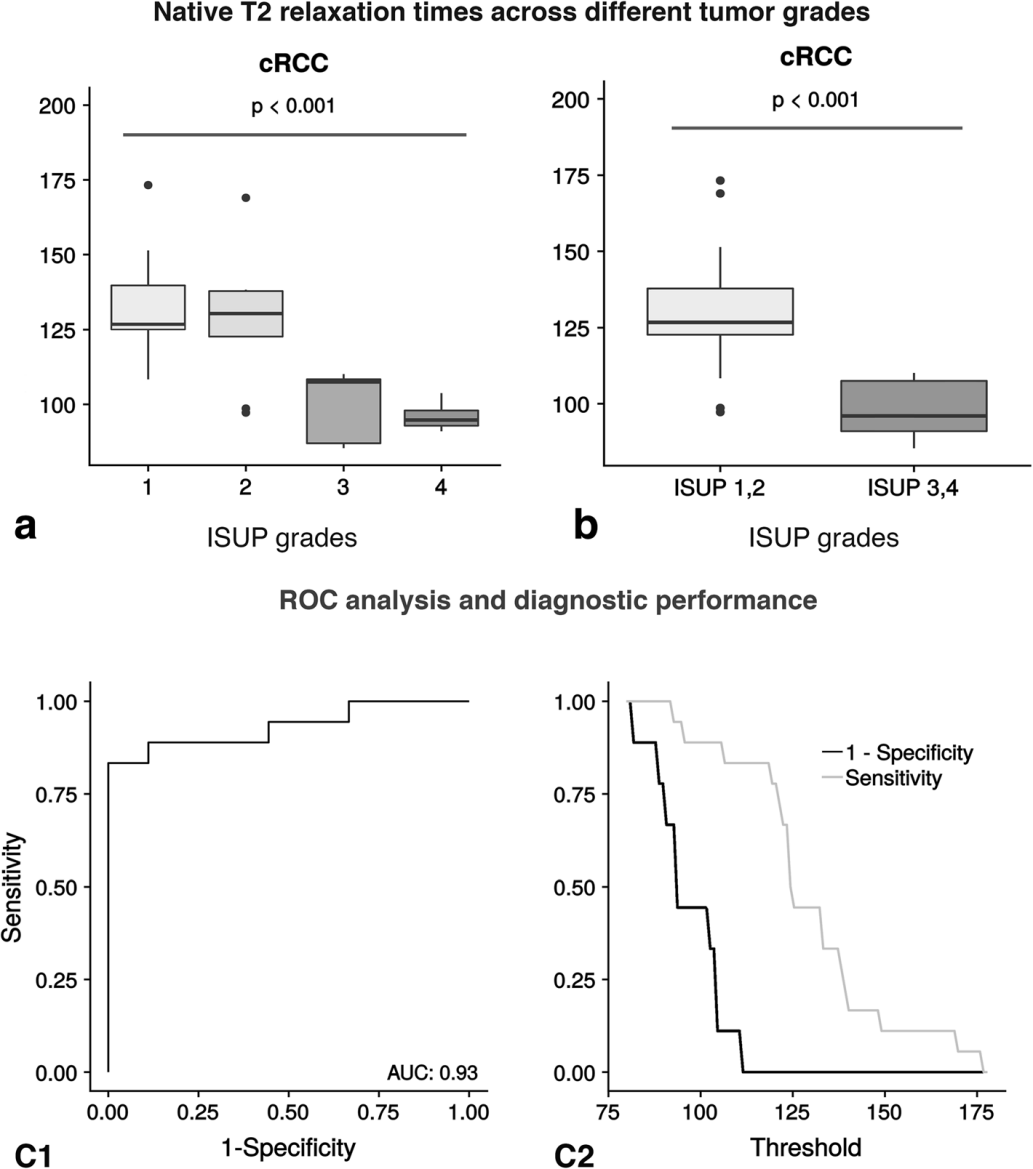
Distribution of T2 across different tumor grades (ISUP grades). The upper left part of the Fig. **a** displays the T2 differences across four different ISUP grades using boxplots. And the upper right part of the Fig. **b** shows the T2 differences across a two-tier-system (ISUP 1,2 against ISUP 3,4). Lower grade cRCC show higher T2 values compared to higher grade cRCC. The lower left part of Fig. **C1** illustrates the diagnostic performance of T2 mapping as a binary classifier in discriminating between ISUP grades 1–2 and 3–4. In this context, the T2 threshold is varied using a receiver operation characteristic curve (ROC-curve). The corresponding Area under the Curve (AUC) is 0.93. The lower right part of the Fig. **C2** displays the respective sensitivity and specificity values plotted against their corresponding threshold. The centerline in each box represents the median, whereas the lower and upper limits of each box represent the 25th and 75th percentiles, respectively. Whiskers extend to the most extreme observations within 25th and 75th percentiles ±1.5 x interquartile range. Observations outside these whiskers are shown as dots

**Table 2:** Characteristics of the Study Population

Number of patients with cRCC (men, percentage of total (%))	27 (19, 70.4)
Mean age of patients with cRCC ± SD^a^	61.3 ± 14.2
Median diameters for cRCC (IQR^b^)	
*ISUP grade 1* (IQR)	3.5 (1.13)
*ISUP grade 2* (IQR)	3.95 (3.3)
*ISUP grade 3* (IQR)	15.5 (3.8)
*ISUP grade 4* (IQR)	5.6 (2.7)
Partial nephrectomy (number, %)	15, 55.6
Radical nephrectomy (number, %)	10, 37.0
Biopsy (number, %)	2, 7.4
Imaging Characteristics.	
Average normal renal parenchyma T2 values (ms) ± SD	
*Renal cortex*	85 ± 16
*Renal medulla*	92 ± 16
Average T2 values for cRCC (ms) ± SD (number, %)	
*ISUP grade 1* ± SD	134 ± 20 (8, 29.6)
*ISUP grade 2* ± SD	128 ± 21 (10, 37.0)
*ISUP grade 3* ± SD	108 ± 19 (5, 18.5)
*ISUP grade 4* ± SD	96 ± 6 (4, 14.8)

^a^*SD* Standard deviation, ^b^*IQR* Interquartile range

Histologic classification of patients revealed 8 ISUP grade 1 lesions, 10 ISUP grade 2 lesions, 5 ISUP grade 3 lesions, and 4 ISUP grade 4 lesions. The maximum cRCC diameter as determined in T2 HASE images, using the longest tumor diameter in coronal sections, was between 1.4 cm and 17 cm (median of 4, interquartile range of 4.7). There was no difference in tumor size between men and women (*p* = 0.21). The interval between MRI imaging and surgical removal was 25.1 ± 20.7 days.

### T2 mapping results for different tumor grades

**Fig. 3:**
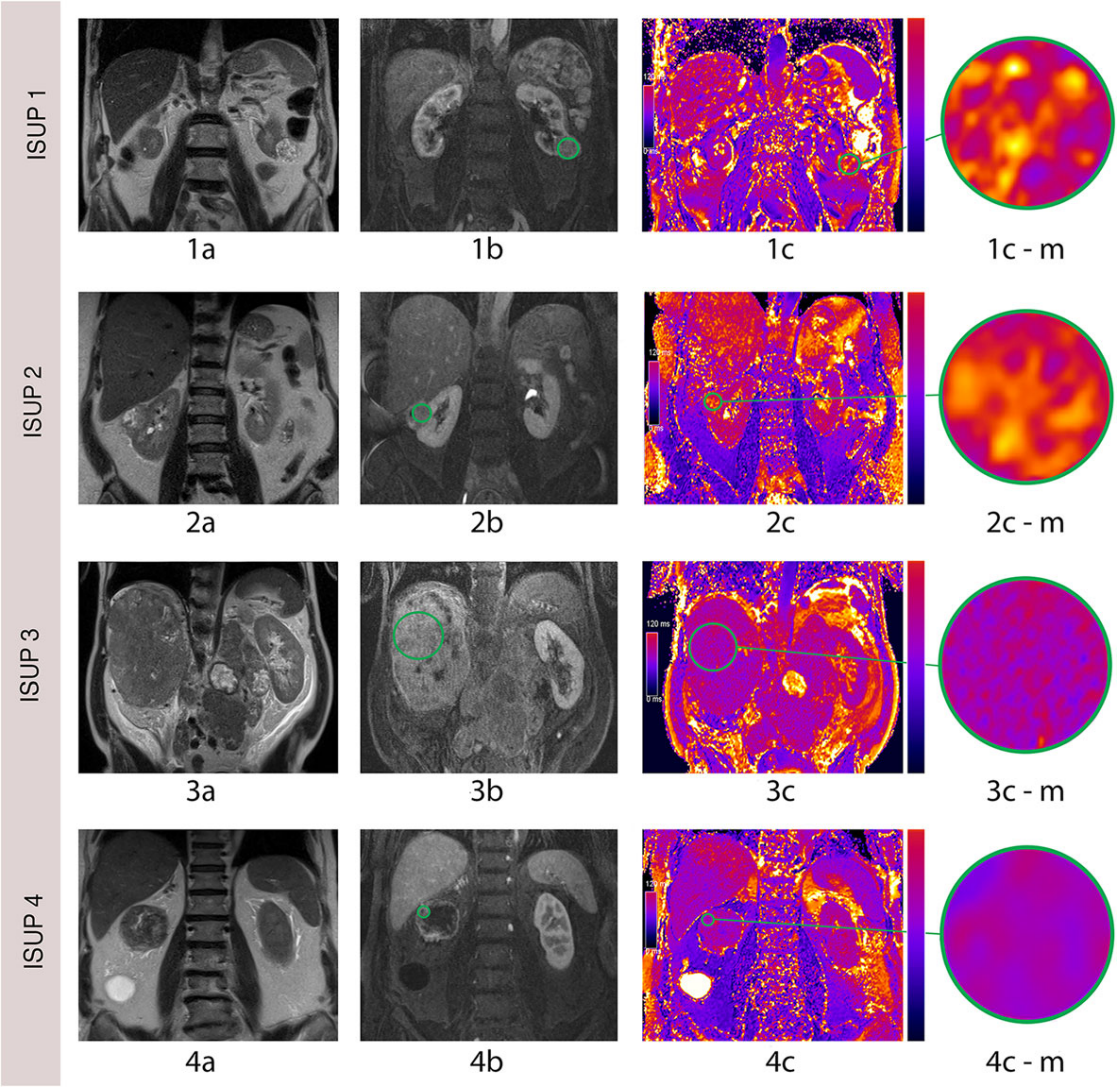
Exemplary T2 mapping images of lower and higher grade cRCC. 1a, coronal T2 HASTE image of a 77-year-old man with a low grade (ISUP 1) cRCC of the left kidney. 1b, postcontrast T1 FLASH image. 1c, corresponding TrueFISP image, showing a high T2 signal. 2a, T2 HASTE image of a 57-year-old woman with a lower grade (ISUP 2) cRCC of the right kidney. 2b, postcontrast T1 FLASH image. 2c, corresponding TrueFISP image, also showing a high T2 signal (2d). 3a, coronal T2 HASTE image of a 62-year-old man with a higher grade (ISUP 3) cRCC of the right kidney. 3b, postcontrast T1 FLASH image. 3c, corresponding TrueFISP image, showing a low T2 signal. 4a, coronal T2 HASTE image of a 71-year-old man with a high grade (ISUP 4) cRCC of the right kidney. 4b, postcontrast T1 FLASH image. 4c, corresponding TrueFISP image, showing a low T2 signal. 1c-m1, 2c-m1, 3c-m1 and 4c-m1 are magnifications of 1c, 2c, 3c and 4c

The distribution of native T2 relaxation times across different tumor grades can be seen in Fig. 2. Exemplary T2 maps of cRCC patients with different ISUP grades are shown in Fig. 3. T2 relaxation times were higher in lower grade cRCC compared to higher grade cRCC (132 ± 22 ms versus 97 ± 12 ms), with statistical analysis confirming a statistically significant difference (*p* < 0.001). We also looked at the distribution of T2 values in the tumor area based on a whole-tumor-approach, using density plots (refer to Additional file 2: Figure S2 and Additional file 3: Figure S3.

Average T2 values were 134 ms ± 20 ms for ISUP grade 1, 128 ms ± 21 ms for ISUP grade 2, 108 ms ± 19 ms for ISUP grade 3 and 96 ms ± 6 ms for ISUP grade 4 tumors. For the normal renal cortex and medulla, the average T2 values were 92 ± 16 and 85 ± 16, respectively. There was no significant correlation between values for renal medulla, cortex and the glomerular filtration rate (r = 0.21, *p* = 0.89).

### Diagnostic performance of T2 mapping

A ROC analysis was performed to determine the optimal T2 cut-off-value for the distinction of lower grade from higher grade cRCC. A cut-off-value of 110 ms could be identified to detect lower grade cRCC with a sensitivity of 83.3% (95% CI: 0.59–0.96) and a specificity of 88.9% (95% CI: 0.52–1.00) (refer to Table 3 for confusion matrix). However, four cases were wrongly diagnosed based on T2 mapping: Three lower grade cRCC were instead classified as higher grade cRCC based on their T2 values. Therefore, based on MRI, the severity of the case was overestimated. And one higher grade cRCC was wrongly classified as lower grade tumor. In this case, MRI underestimated the severity of the case.

**Table 3:** Confusion matrix for the calculation of diagnostic accuracy

MR native T2 mapping (index test)	Pathology (reference standard)		Total
	Confirmed lower grade cRCC	Confirmed higher grade cRCC	
Lower grade cRCC (ISUP grades 1, 2)	15	1	16
Higher grade cRCC (ISUP grade 3, 4)	3	8	11
Total	18	9	27

### Intraobserver and interobserver agreement

**Fig. 4:**
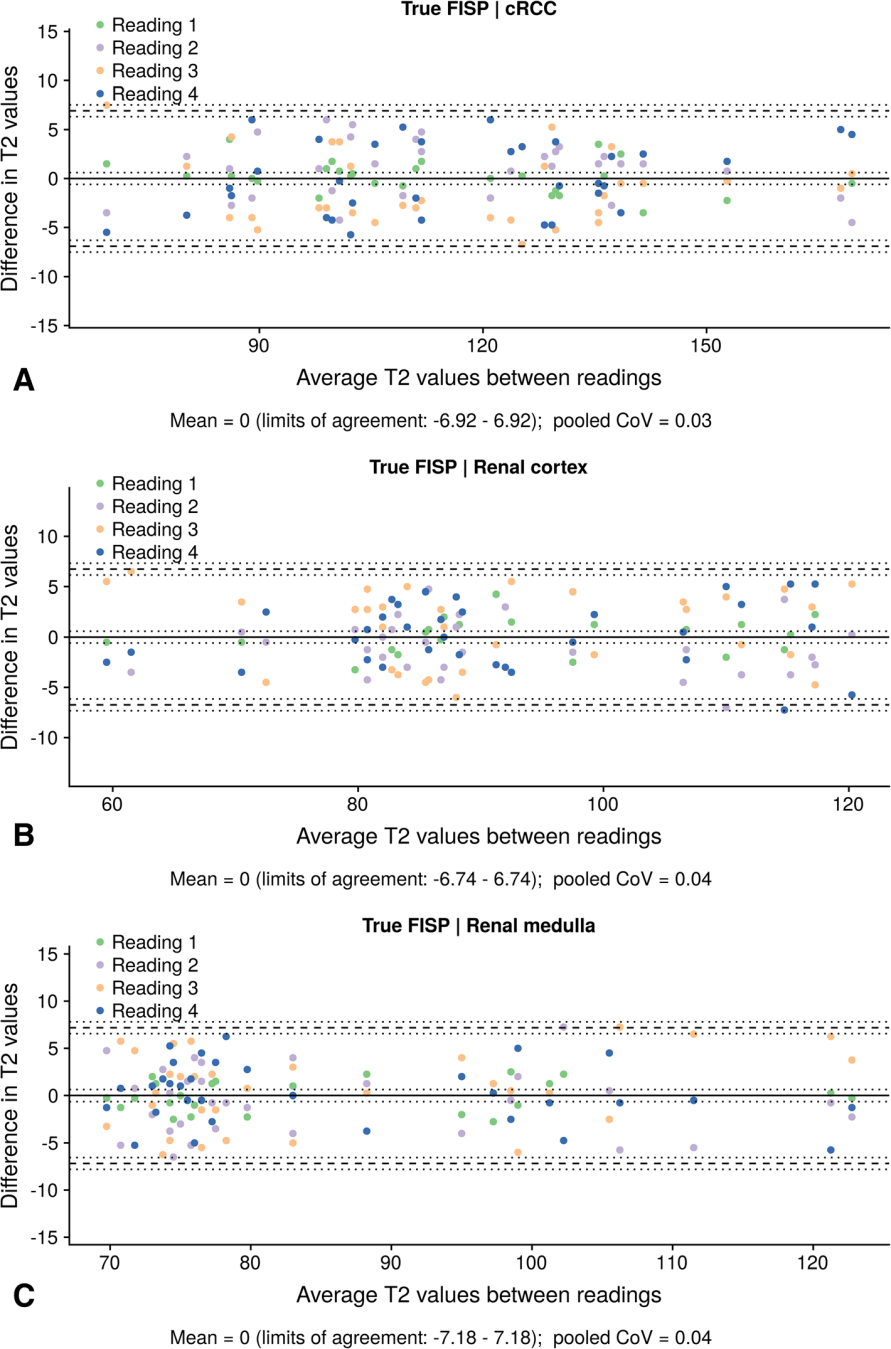
Intraobserver agreement of cortex, medullary and tumor T2 values. Bland-Altman plots, showing agreement between four different readings for measurement of T2 values (**a**, tumor tissue; **b**, renal cortex; **c**, renal medulla). The upper and lower reference lines indicate the upper and lower limits of agreement (95% confidence intervals). The confidence intervals for the mean and limits of agreement are indicated by the dotted lines. CoV: Coefficient of variation

**Fig. 5:**
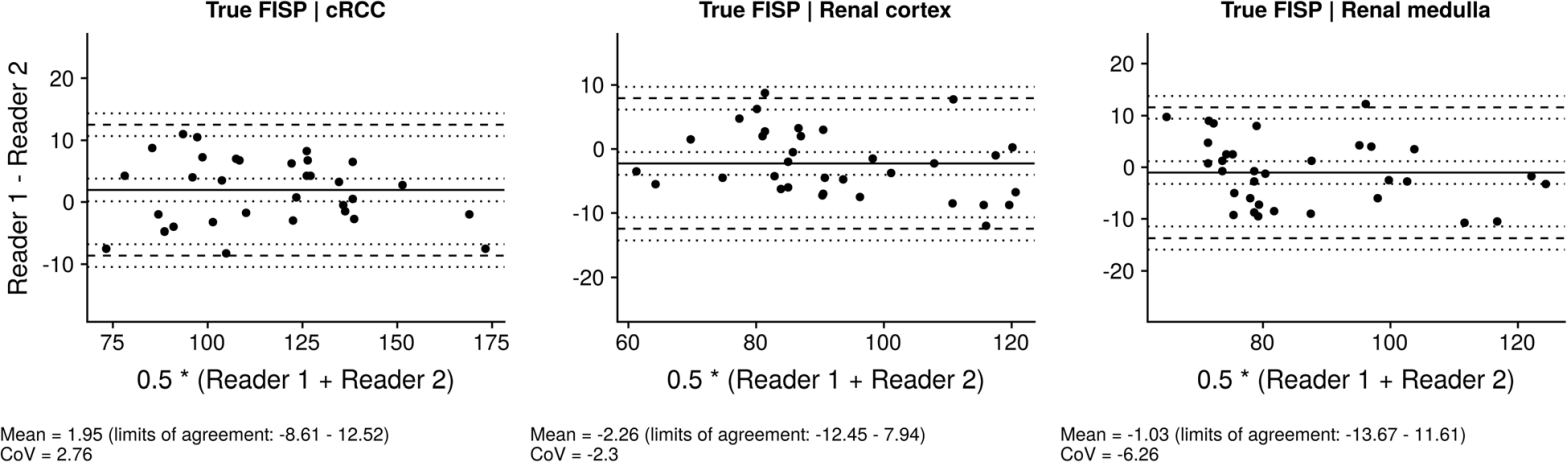
Interobserver agreement of cortex, medullary and tumor T2 values. Interobserver agreement of the cortex, medullary and tumour T2 values. Bland-Altman plots illustrate the interobserver variability for native cRCC, medullary and cortex T2 values. Specifically, the mean differences were 1.95 (95% confidence interval (CI): − 8.61 – 12.52) for the cRCC tumor area with a CoV of 2.76, − 2.26 (95% CI: − 12.45– 7.94) for the renal cortex with a CoV of − 2.3 and − 1.03 (95% CI: − 13.67 – 11.61) for the medullary pyramids with a CoV of − 6.26. The mean difference of the data is illustrated by the central horizontal line. The upper and lower reference lines indicate the upper and lower limits of agreement. The confidence intervals for the mean and limits of agreement are indicated by the dotted lines. CoV: Coefficient of variation

Regarding intraobserver assessment, the confidence intervals with a line of zero difference were 0 (95% CI: − 6.92 – 6.92) for cRCC with a Coefficient of Variation (CoV) of 0.03, 0 (95% CI: − 6.74 – 6.74) for the renal cortex with a CoV of 0.03 and 0 (95% CI: − 7.18 – 7.18) for the medullary pyramids with a Coefficient of Variation (CoV of 0.03 (refer to Fig. 4). For interobserver assessment, the mean differences between two observers were 1.81 (95% CI: − 10.24 – 13.87) for cRCC, − 2.5 (95% CI: − 14.34 – 9.34) for the renal cortex and − 0.59 (95% CI: − 13.17 – 11.98) for the medullary pyramids (refer to Fig. 5). Corresponding to this, there was an excellent interobserver agreement for the cRCC T2 values (ICC 0.97, 95% CI 0.94–0.99), the medullary T2 values (ICC 0.92, 95% CI 0.85–0.96), and the cortex T2 values (ICC 0.94, 95% CI 0.88–0.97).

## Discussion

In this cross-sectional study, a quantitative T2 mapping technique (TrueFISP) was used for the differentiation of lower and higher grade cRCC. Lower grade cRCC (ISUP grades 1, 2) showed significantly higher T2 values compared to higher grade cRCC (ISUP grades 3, 4), supporting the potential of T2 mapping as a noninvasive marker of cRCC grade.

While conventional, qualitative T2 weighted imaging is subject to various limitations, such as the use of arbitrary signal intensity scales for T2 values with subsequent test-retest and interobserver variability, T2 mapping offers the potential for a standardized, reproducible assessment of tissue composition and water contents, e.g. facilitating the diagnosis of interstitial edema and extracellular space expansion [22, 23]. It is based on the acquisition of multiple T2-weighted images at different echo times (TE), with the signal of the various TEs being fitted to an exponential signal decay model, generating an estimate of quantitative T2 values [24]. Direct quantification of the T2 signal provides a benefit over T2-weighted imaging, minimizing dependency on user-defined parameters and subjective interpretation. Besides, differences between tissues might be detected more easily [7]. In the context of cardiac imaging, T2 mapping offers advantages over conventional T2 weighted imaging, for example in the detection of global myocardium changes in myocarditis or cardiac allograft rejection [25, 26]. For oncological imaging, T2 mapping was previously used for a range of different malignancies, such as colorectal carcinoma, breast and ovarian cancer, prostate carcinoma, glioblastoma and brain metastases [27–32]. Only a handful of prior studies focused on renal T2 mapping, e.g. showing the potential to evaluate the renal parenchyma after kidney transplantation and to improve the diagnosis/progression of polycystic kidney disease [11, 33]. Medullary T2 relaxation times were observed to be consistently longer compared to cortical T2 relaxation times, which is in accordance with the results of the present study [13]. To our knowledge, T2 mapping has so far not been examined in the context of cRCC.

Non-invasive cRCC grading would not only improve preoperative planning options, but also aid in prognosis assessment and prior patient information, for example by informing patients about the best individual therapy options. Furthermore, it may reduce the need for kidney mass biopsies. In addition, it might prevent cases of upstaging after partial nephrectomy in larger tumors, help identify patients who might be eligible for immunotherapy, and, finally, facilitate the selection of patients suitable for less invasive therapies [27], including minimally invasive procedures such as ablation, cryotherapy or even active surveillance. In addition to image-based grading of cRCC, image-based identification of different renal tumor subtypes such as urothelial carcinomas, oncocytomas, chromophobic RCC or lipid-poor angiomyolipomas would also be highly beneficial, because it could reduce the number of unnecessary surgical resections (9, 39). In the present study, however, the number of subtypes other than cRCC was too small to allow a valid evaluation (3 urothelial carcinomas, 2 oncocytomas, 2 atypical angiomyolipomas). Further studies are warranted to better assess the clinical feasibility of T2 mapping for the noninvasive assessment of cRCC, focusing on the ability to predict both tumor grade and subtype.

Regarding histology, cRCC consists of cells with clear cytoplasm and necrosis with often occurring cystic degeneration or hemorrhage [34]. Lower grade cRCC are predominantly characterized by cystic changes, which could serve as an explanation for the observed prolonged relaxation times. Necrosis, on the other hand, is particularly common in higher grade cRCC [6]. It does not only occur as macroscopic necrosis, but also as micronecrosis below the spatial resolution of MRI [6, 35, 36]. In the present study, false negative results (lower grade cRCC with T2 values below 110 ms) could thus have resulted from including areas of (micro)necrosis within the ROI analysis [37, 38]. Decreased T2 relaxation times in higher grade tumors may be based on the presence of densely packed proliferating cells, interstitial reticulin deposition and/or irregular tumor vasculature [39, 40]. The false positive result in our study could have resulted from the inclusion of (micro)cystic components.

Although a considerable difference in the average T2 relaxation times between lower grade and higher grade cRCC was observed in a two-tier-system, there was a significant overlap between the subgroups (ISUP 1,2 and ISUP 3,4) with no apparent difference. As a consequence, based on our data, it was not possible to differentiate between ISUP grade 1 and 2 or ISUP grade 3 and 4 tumors. However, the number of patients in the subgroups, especially regarding higher grade tumors, was very small and future studies with larger case numbers might help to assess the feasibility of T2 mapping in a four-tier-grading system, potentially allowing for/leading to T2 mapping value reference ranges with diagnostic utility. On route towards renal T2 mapping as a potential additional biomarker, it will, furthermore, be necessary to validate T2 mapping against accepted reference measurements, such as nuclear medicine evaluations as well as histological findings [13].

In the context of future clinical applications, the use of T2 mapping might be a helpful addition to multiparametric imaging instead of being used exclusively. To this end, a novel T2-mapping-derived parameter could be integrated into a multiparametric MR imaging model. In the context of radiomics, it could also be included as a quantitative imaging feature, be converted into minable data and aid in the building of predictive models. It can be expected, that multiparametric approaches will yield superior diagnostic performance, when compared to single imaging parameters alone. Therefore, in the future, multiparametric approaches may enable reliable, noninvasive grading of cRCC.

Regarding practicability, the T2 mapping approach chosen in the present study would be easy to implement in a clinical setting, as no complex mathematical modelling is required and the TrueFISP sequence is already commercially available on some MR scanners. Furthermore, it enables the acquisition of T2 maps during MRI scanning with a short scanning time. TrueFISP has been previously shown to be a robust method for T2 mapping, combining T2-magnetization preparation with steady-state free precession imaging and being sufficiently fast to be acquired in a single breathhold [41]. An advantage of T2-prepared single-short TrueFISP is its high signal-to-noise-ratio (SNR) and low sensitivity to breathing artifacts, which can be especially helpful in the assessment of cRCC, as kidney imaging is often limited by respiratory motion artefacts because of the location in the upper abdomen [7, 42]. Potential disadvantages of TrueFISP result from the fact, that the number of acquired echoes is considerably lower compared to other techniques such as Multi Echo Spin Echo (MESE), providing only a limited number of data points along the T2 decay curve and thus potentially compromising accuracy and limiting its use to a narrower range of T2 species [43].

Due to the relatively long T2 relaxation times of the kidneys, ideally longer T2 preparation times with more than three weightings are used. While a repetition time of 1000 ms allows for time-efficient acquisition within a single breathhold, it does not ensure complete signal recovery. Therefore, the T2 maps in the present study are partly influenced by renal T1 signal and do not correspond to a pure T2 signal. To overcome this limitation in future studies, either the time of repetition can be increased (with a subsequent increase in scan time) or the flip angle can be reduced (with a decrease in SNR). However, within the scope of the present proof, we first aimed to investigate if a cardiac T2 mapping approach would be feasible for kidney imaging at all. Another limitation of this proof-of-concept study is, that it was based on a single-center design, only measuring T2 values at one time point prior to the intervention/surgery and including a small number of patients, especially with regard to higher grade cRCC. The small number of images was also an important limitation for assessment of intraobserver and interobserver agreement. In addition, the exclusion of other subtypes of renal masses brings about the risk of potential selection bias. Also, even though larger areas of necrosis were excluded, smaller necrotic regions may have been included in the ROI measurements and might have affected T2 measurements. Furthermore, the T2 relaxation time of the tumor was only measured in one representative coronal plane, not in the whole tumor. Besides, no automatic motion or alignment correction was applied to correct for misregistrations between the different sequences. Therefore, slight misalignments cannot be completely excluded. Apart from that, even though patients were advised not to thirst and to drink water in the morning before the MRI, this was not explicitly controlled prior to the examination and patients were not instructed to drink a specific amount of water. Therefore, patients may have exhibited certain fluctuations in the hydration status, which could have affected T2 measurements in the renal parenchyma and potentially also in the tumor tissue. Finally, only one scanner from one vendor was used, therefore the results may not be generalizable to other institutions. Further investigations will be required to validate T2 mapping as a tool for cRCC assessment.

## Conclusions

There was a significant difference in average T2 relaxation times between lower grade and higher grade cRCC, with lower grade cRCC showing significantly longer T2 relaxation times compared to higher grade cRCC. We therefore believe, that this technique holds potential for the future to noninvasively assess cRCC tumor grade in vivo and could provide a helpful addition to multiparametric imaging. However, studies with larger patient cohorts and a broader range of higher grade tumors are required to explore the utility of T2 mapping as a possible primary diagnostic MRI sequence for cRCC grading.

## Additional files

Additional file 1: **Figure S1.** Visualization of circular 2D ROI placement for smaller tumors without and larger tumors with apparent necrosis zones for ISUP grades 1 to 4. ROIs were first placed in a corresponding postcontrast image and then copied to the T2 map. (TIF 8355 kb)

Additional file 2: **Figure S2.** Segmentation of tumors (ISUP grades 1 to 4 from top to bottom, A-D) with creation of image masks (A2 through D2). A3 through D3 show the calculated percentage densities of the absolute T2 values. (TIF 2244 kb)

Additional file 3: **Figure S3.** The upper part of the figure illustrates the percentage density of T2 values for whole-tumor measurements of the four colour-coded ISUP grades (refer to the legend on the upper right side). For each tumor, the T2 maps were segmented and image masks were imported into the open access software ‘R’. The lower part of the figure shows the colour-coded percentage density of T2 values for lower grade tumors (combined ISUP grades 1 and 2) and higher grade tumors (combined ISUP grades 3 and 4). (TIF 773 kb)

## Data Availability

The datasets used and/or analysed during the current study are available from the corresponding author on reasonable request.

## Abbreviations

CoV: Coefficient of Variation
cRCC: Clear-cell renal cell carcinoma
ICC: Intraclass correlation coefficient
ISUP: International Society of Urological Pathology
MRI: Magnetic resonance imaging
RCC: Renal cell carcinoma
ROC: Receiver Operating Characteristic Curve
ROI: Region of interest
SNR: Signal-to-noise-ratio
TE: Echo time
WHO: World Health Organization
