# Supplementary figures and images for: Use of quantitative T2 mapping for the assessment of renal cell carcinomas: first results
Source: Cancer Imaging. 2019 Jun 7;19:35. doi: 10.1186/s40644-019-0222-8 (PMC6555952; doi:10.1186/s40644-019-0222-8)

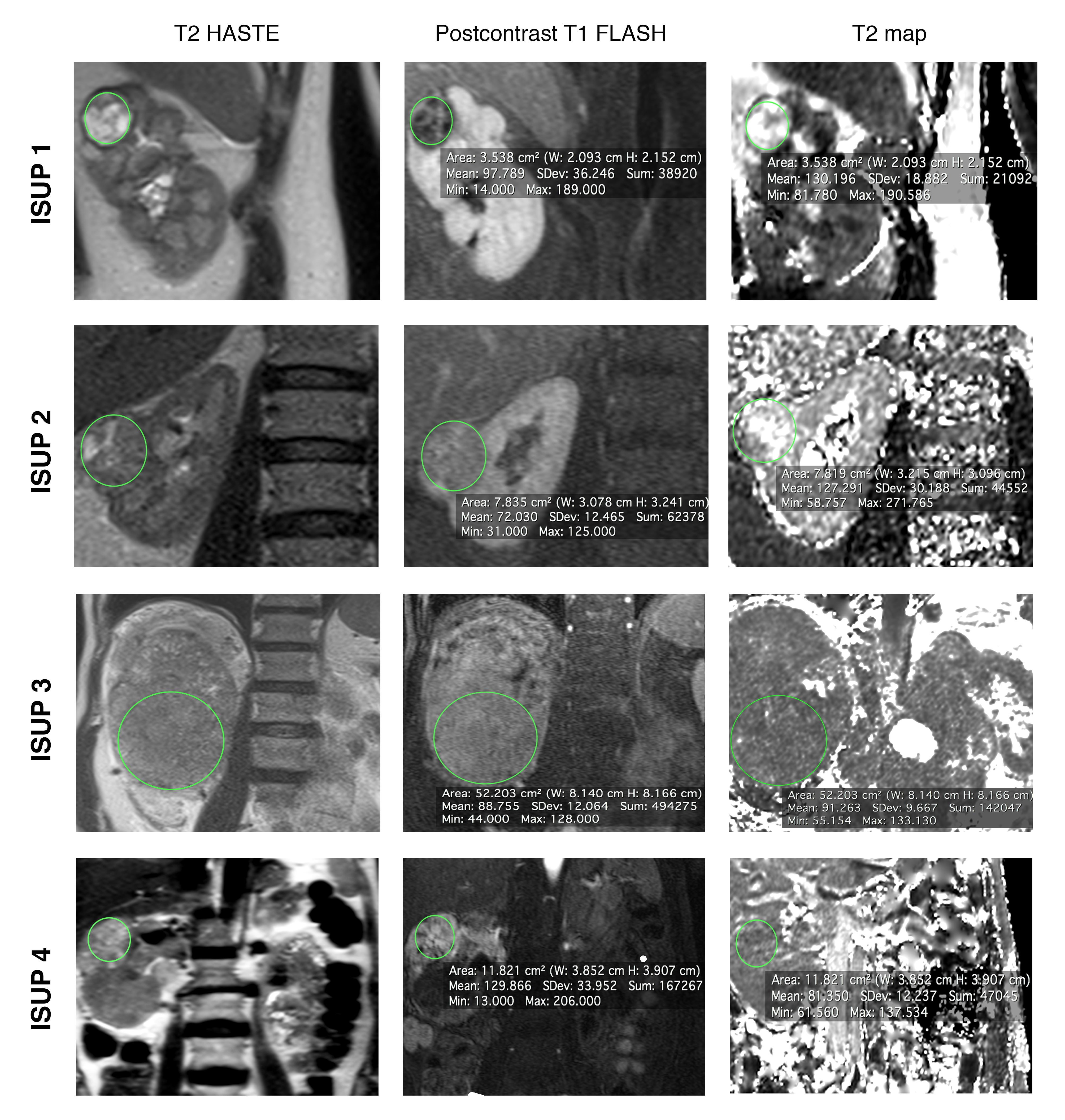

Supplement: Supplementary file 1 — Figure S1. Visualization of circular 2D ROI placement for smaller tumors without and larger tumors with apparent necrosis zones for ISUP grades 1 to 4. ROIs were first placed in a corresponding postcontrast image and then copied to the T2 map. (TIF 8355 kb) [file 40644_2019_222_MOESM1_ESM.tif]

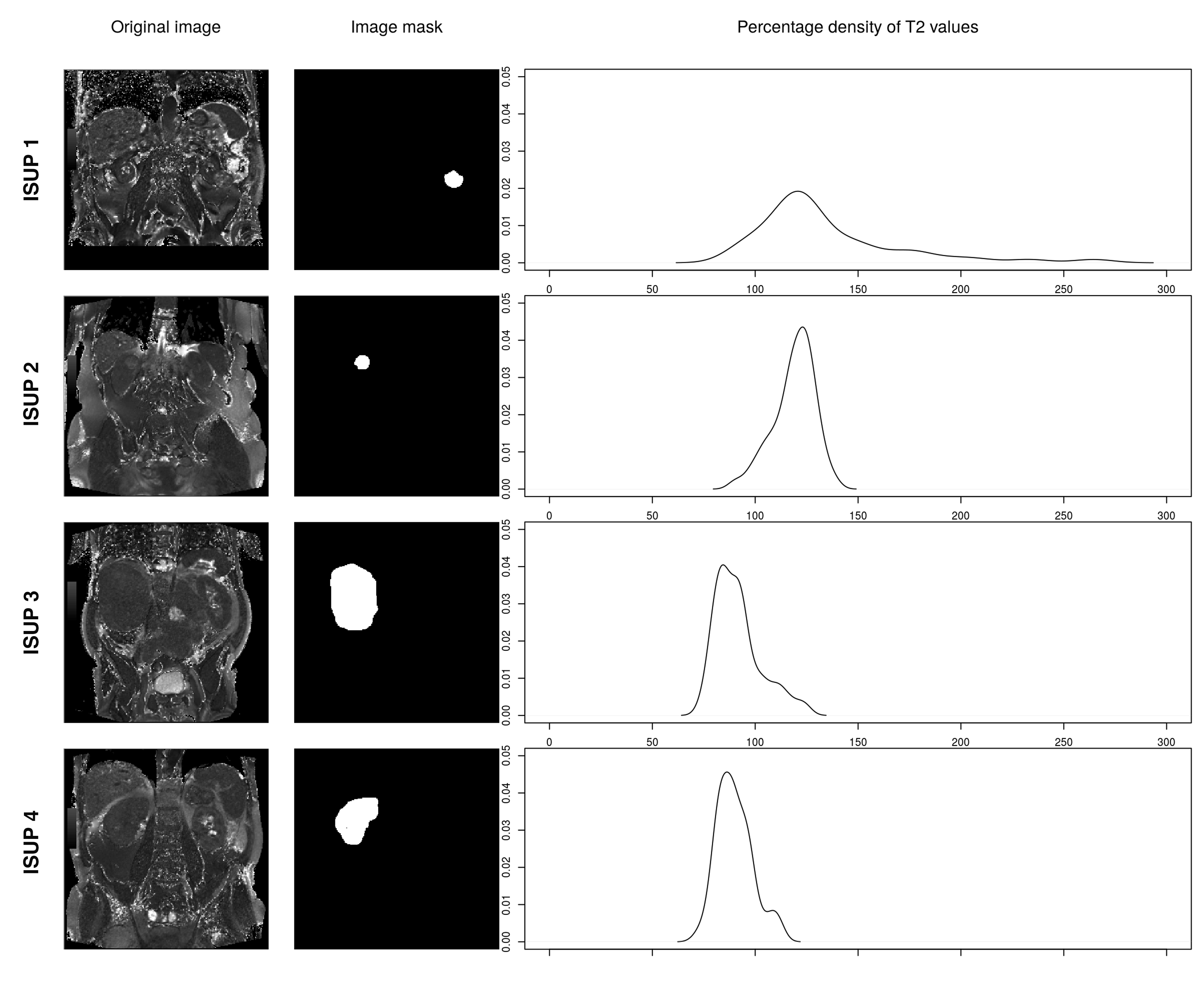

Supplement: Supplementary file 2 — Figure S2. Segmentation of tumors (ISUP grades 1 to 4 from top to bottom, A-D) with creation of image masks (A2 through D2). A3 through D3 show the calculated percentage densities of the absolute T2 values. (TIF 2244 kb) [file 40644_2019_222_MOESM2_ESM.tif]

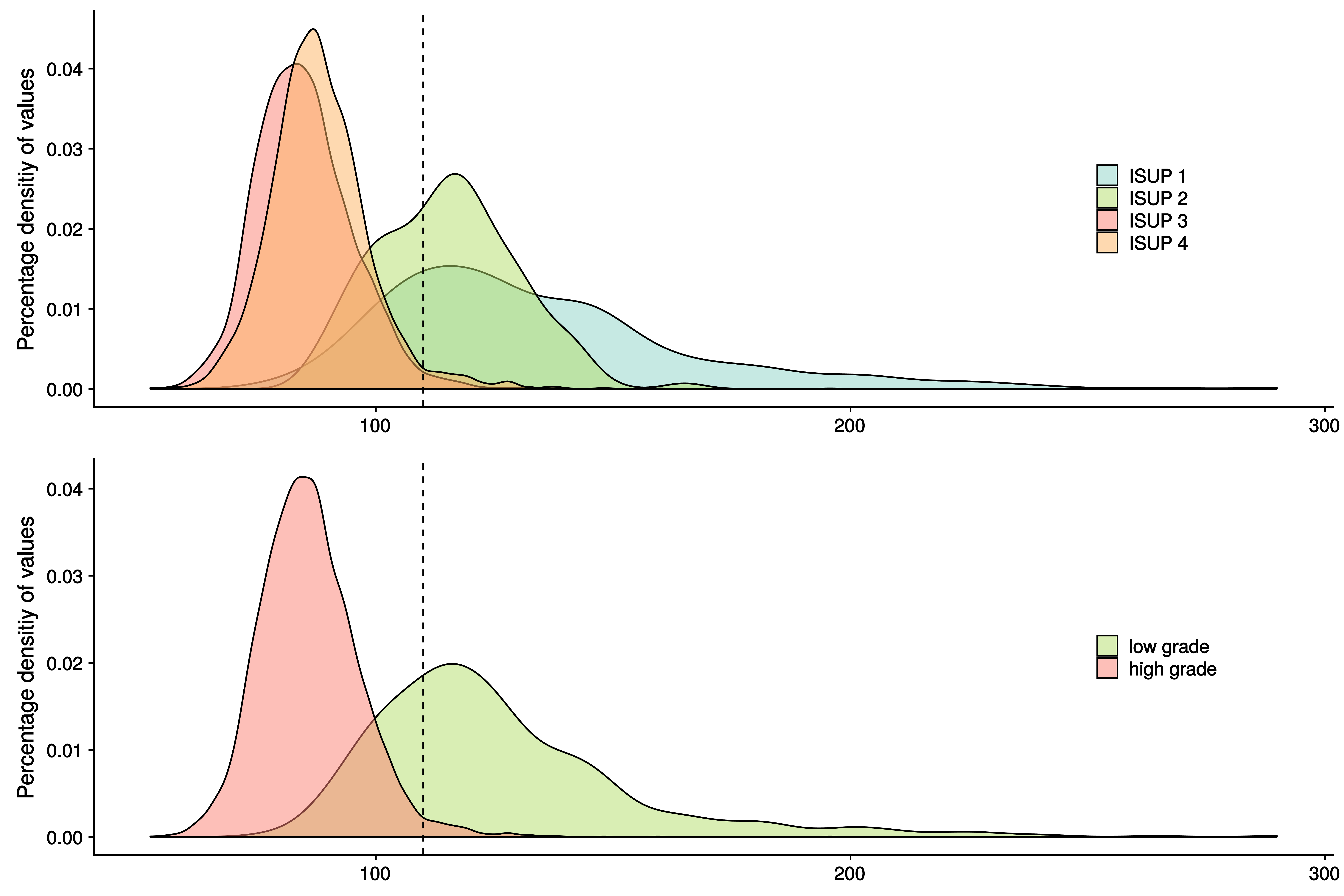

Supplement: Supplementary file 3 — Figure S3. The upper part of the figure illustrates the percentage density of T2 values for whole-tumor measurements of the four colour-coded ISUP grades (refer to the legend on the upper right side). For each tumor, the T2 maps were segmented and image masks were imported into the open access software ‘R’. The lower part of the figure shows the colour-coded percentage density of T2 values for lower grade tumors (combined ISUP grades 1 and 2) and higher grade tumors (combined ISUP grades 3 and 4). (TIF 773 kb) [file 40644_2019_222_MOESM3_ESM.tif]
